# A computational model of cell culture dynamics: the role of connectivity and synaptic receptors in the appearance of synchronized bursting events

**DOI:** 10.1186/1471-2202-16-S1-P177

**Published:** 2015-12-18

**Authors:** Davide Lonardoni, Stefano Di Marco, Hayder Amin, Luca Berdondini, Thierry Nieus

**Affiliations:** 1 Istituto Italiano di Tecnologia, Genova, Italy

## 

How an ensemble of neurons wires together to form a functional unit is a fundamental problem in neuroscience. The architecture of neuronal wiring, in fact, determines how neurons communicate and may be important for information processing performed by neuronal networks. However current knowledge is mainly limited to networks consisting of a small number of neurons, while the topological structure of biological networks remains still unknown. Primary neuronal cultures represent an ideal preparation to investigate the basic principles of network dynamics. At the mature stage, they display network-wide synchronous bursting events (SBEs) sharing similar spatio-temporal patterns of firing [1]. Interestingly, high-density MEA recordings have shown that SBEs actually correspond to propagating activities through the network. Typically, the simulated SBEs originated from a few and specific sites as in experiments [2], but the nature and the role of such events is still under debate. In order to investigate the determinants of such dynamics, we developed a computational model that mimics the main features of the recordings obtained by a high density multi-electrode-array device (4096 electrodes inter-spaced by 20um, [3]). With only a few topological constraints, the model expressed realistic SBEs along time that can be well clustered into only a few groups differing for their ignition sites and propagation directions, similarly to what it is observed experimentally. Furthermore, we used the model together with experimental datasets to investigate the effects of synaptic blockers of the AMPA, NMDA and GABA synapses on the network activity. In particular, we showed that NMDA receptors can be among the principal mechanisms involved in triggering a sequence of SBEs, a firing regime that is typically observed in for mature neuronal cultures. Such regime is characterized by a principal SBE recruiting a great percentage of neurons and followed by a sequence of several weaker SBEs interleaved by hundreds of milliseconds.

Altogether, the results obtained with our neural network computational model show that this model can replicate most of the salient firing properties observed experimentally in cultured neuronal networks and that it can serve for exploring the properties of signals and responses observed in neuronal networks properties.
